# Changes in pacing variation with increasing race duration in ultra-triathlon races

**DOI:** 10.1038/s41598-023-30932-1

**Published:** 2023-03-06

**Authors:** Mirko Stjepanovic, Beat Knechtle, Katja Weiss, Pantelis Theodoros Nikolaidis, Ivan Cuk, Mabliny Thuany, Caio Victor Sousa

**Affiliations:** 1 Institute of Primary Care, University of Zurich, Zurich, Switzerland; 2 Medbase St. Gallen Am Vadianplatz, Vadianstrasse 26, 9001 St. Gallen, Switzerland; 3 School of Health and Caring Sciences, University of West Attica, Athens, Greece; 4 Faculty of Sport and Physical Education, University of Belgrade, Belgrade, Serbia; 5 Faculty of Sports, University of Porto, CIFI2D Porto, Portugal; 6 Health and Human Sciences, Loyola Marymount University, Los Angeles, CA USA

**Keywords:** Physiology, Environmental impact

## Abstract

Despite the increasing scientific interest in the relationship between pacing and performance in endurance sports, little information is available about pacing and pacing variation in ultra-endurance events such as ultra-triathlons. Therefore, we aimed to investigate the trends of pacing, pacing variation, the influence of age, sex, and performance level in ultra-triathlons of different distances. We analysed 969 finishers (849 men, 120 women) in 46 ultra-triathlons longer than the original Ironman® distance (e.g., Double-, Triple-, Quintuple- and Deca Iron ultra-triathlons) held from 2004 to 2015. Pacing speed was calculated for every cycling and running lap. Pacing variation was calculated as the coefficient of variation (%) between the average speed of each lap. Performance level (i.e., fast, moderate, slow) was defined according to the 33.3 and 66.6 percentile of the overall race time. A multivariate analysis (two-way ANOVA) was applied for the overall race time as the dependent variable with ‘sex’ and ‘age group’ as independent factors. Another multivariate model with ‘age’ and ‘sex’ as covariates (two-way ANCOVA) was applied with pacing variation (cycling and running) as the dependent variable with ‘race’ and ‘performance level’ as independent factors. Different pacing patterns were observed by event and performance level. The general pacing strategy applied was a positive pacing. In Double and Triple Iron ultra-triathlon, faster athletes paced more evenly with less variation than moderate or slower athletes. The variation in pacing speed increased with the length of the race. There was no significant difference in pacing variation between faster, moderate, and slower athletes in Quintuple and Deca Iron ultra-triathlon. Women had a slower overall performance than men. The best overall times were achieved at the age of 30–39 years. Successful ultra-triathlon athletes adapted a positive pacing strategy in all race distances. The variation in pacing speed increased with the length of the race. In shorter ultra-triathlon distances (i.e., Double and Triple Iron ultra-triathlon), faster athletes paced more evenly with less variation than moderate or slower athletes. In longer ultra-triathlon distances (i.e., Quintuple and Deca Iron ultra-triathlon), there was no significant difference in pacing variation between faster, moderate, and slower athletes.

## Introduction

An ultra-endurance performance is defined as any endurance performance of six hours in duration or longer^1^. A triathlon is characterized by the successive completion of three disciplines (e.g., swimming, cycling, and running). Therefore, an Ironman® triathlon with 3.8 km swimming, 180 km cycling, and 42.2 km running must be considered an ultra-endurance performance considering the men’s world record being at 7:21:12 h:min:s^2^.

In addition to the single Ironman® distance, ultra-triathlon races of x-times the Ironman® distance exist, such as the Double Iron ultra-triathlon (7.6 km swimming, 360 km cycling and 84 km running), the Triple Iron ultra-triathlon (11.4 km swimming, 540 km cycling and 126.6 km running), the Quintuple Iron ultra-triathlon (19 km swimming, 900 km cycling and 211 km running), and the Deca Iron ultra-triathlon (38 km swimming, 1800 km cycling and 422 km running). The popularity of ultra-triathlons is increasing, indicated by a rising number of participants^3–7^. For example, participation trends during 1985–2009 showed an increase in both Double and Triple Iron (i.e., 17–98 per year in Double and 7–41 per year in Triple Iron, respectively)^8^. Similar results were shown by Sigg et al., in which the number of triathletes from Europe has increased for both sexes, while the numbers for North America increased only for the woman athletes^9^.

An important aspect regarding performance in endurance events is the distribution of exercise intensity. Athletes need to distribute their metabolic energy as fuel for their activity to avoid premature fatigue by going too fast early on^10^. This has been often termed as ‘pacing’^11,12^. There are six different pacing strategies such as positive pacing (i.e., slowing over time), negative pacing (i.e. increase in speed over time), all-out pacing (i.e., maximal speed possible), even pacing (i.e. same speed over time), variable pacing (i.e., pacing with multiple fluctuations) and parabolic-shaped pacing (i.e., positive and negative pacing in different segments of the race)^13^. Research regarding pacing has increased over the past years^10–12,14–20^. Positive pacing has been shown as the adequate strategy adapted in many races and disciplines (i.e., for elite Ironman® triathletes, ultra-cyclists, and swimmers)^21–23^.

Race distance should be considered a factor that influences pacing strategy^13^. It has been shown that athletes pace positively in long and ultra-long races^22^. Considering triathlon, in both an Olympic distance and an Ironman® 70.3 distance triathlon, pacing strategies were strongly influenced by both the distance and the discipline^17^. Regarding longer triathlon distances, finishers of a one per day Deca Iron ultra-triathlon applied positive pacing^20^. At the ‘Ultraman Hawaii’, women paced differently than men (i.e. applied a more even pace after increasing speed at the start of the race)^16^. However, no study systematically investigates the effect of race distances on pacing strategies applied in very long triathlon distances such as Double Iron-, Triple Iron-, Quintuple Iron- and Deca Iron ultra-triathlon.

Another aspect is the performance level, where faster finishers pace differently than slower finishers and elite athletes pace differently than recreational athletes^24–26^. In ultra-endurance running such as 100-km ultra-marathon running, faster runners had fewer changes in running speed and started at a higher running speed while maintaining running speed longer^27^. Among marathoners, slower finishers showed a greater pace variability than faster finishers^26^. To the best of our knowledge, there is no study investigating the influence of performance level on pacing in ultra-triathlon.

Several physiological and psychological factors can influence both performance and pacing during endurance and an ultra-endurance performance, such as age^5^, sex^16^, race distance^11^, performance level^28^, mechanical damage to muscle fibers^29^, increasing body temperature^30^, reduction in neuromuscular activity^31^ and muscle glycogen depletion^8^.

Additionally, gender seems to influence pacing during a triathlon race^32,33^. In elite Ironman® triathletes, women were significantly slower when applying the same positive pacing strategy^15^. However, at the ‘Ultraman Hawaii’, a multistage event consisting of 10 km swimming, 165 km cycling (day 1), 261 km cycling (day 2) and 85 km running (day 3), women applied a more even pacing strategy compared to men. The fastest women decreased performance on day 1 and could then maintain on days 2 and 3, whereas the fastest men impaired performance on days 1 and 2 but improved on day 3^16^. A comparison of top performers from the Ironman® to the Double Deca Iron ultra-triathlon distance showed that men were faster than women and that the sex difference increased for swimming, running, and overall race time but not for cycling^34^. There is, however, no study investigating all finishers (i.e., not only the top athletes).

Age has an influence on endurance performance and pacing. Regarding triathlon, performance and age-related trends in elite triathletes competing in age group classes have been investigated for an Ironman® triathlon such as ‘Ironman® Hawaii’^16,35^ and short distances such as the Olympic distance triathlon^36^. An age-related performance decline has been shown for all investigated distances^37^. In Ironman® triathletes, the age-related performance decline started at a higher age than in short distance triathletes^3^.

Another important aspect is the age of peak performance. When Olympic, Ironman® 70.3 and Ironman® distance races were compared, the age of peak triathlon performance was higher in the longer triathlon race distances^38^. For ultra-triathlon, Deca Iron ultra-triathletes were older than Triple Iron ultra-triathletes^39^. However, the age-related performance and the age of peak performance for the Double and Quintuple Iron ultra-triathlon distance are unknown.

Based on the limited existing literature regarding the effect of the race distance, age, gender, and performance level on pacing in ultra-triathlon races we hypothesized, firstly, that a positive pacing strategy would be applied on the shorter distances (e.g., Double and Triple Iron ultra-triathlon) and an even pacing on the longer ones (e.g., Quintuple and Deca Iron ultra-triathlon). Secondly, we hypothesized that slower finishers apply more variable pacing. Thirdly, we hypothesized to find differences in pacing between men and women. We hypothesized that the age-related declines would occur later at the longer distances (e.g., Quintuple and Deca Iron ultra-triathlon) compared to the shorter distances (e.g., Double and Triple Iron ultra-triathlon).

## Materials and methods

### Ethical approval

The Institutional Review Board of Kanton St. Gallen, Switzerland approved all procedures used in the study with a waiver of the requirement for informed consent of the participants given the fact that the study involved the analysis of publicly available data (01/06/2010). The study was conducted in accordance with recognized ethical standards according to the Declaration of Helsinki adopted in 1964 and revised in 2013.

### Subjects

We focused on distances longer than the original Ironman® distance, like the Double Iron, Triple Iron, Quintuple Iron and Deca Iron ultra-triathlon. Overall results are available on the International Ultra Triathlon Association homepage (IUTA, https://www.iutasport.com/). We contacted all race directors directly for the lap times in running, cycling and for the contestants’ age. Lap times were provided electronically in in spreadsheets. Furthermore, the age of all finishers was provided. The athletes were sorted into age groups of 10-year age intervals. Lap distances were different for each race as every race has its course. Tables 1 and 2 provide an overview of the number of finishers for each race. We excluded all non-finishers. The number of calculated laps for each race type was provided additionally.

**Table 1:** Number of finishers for each race type and number of excluded finishers.

	Races	Double	Triple	Quintuple	Deca	Excluded	Total
	Male	346	499	23	23	42	849
Finishers	Female	58	52	6	6	2	120
	Overall	404	551	29	29	44	969
Laps	Bike	22,184	38,334	3560	5745		69,823
	Run	19,231	51,404	3311	9818		83,764

**Table 2:** All races included in the statistical analysis sorted by race type and year.

Double		Triple		Quintuple		Deca	
2014	Virginia (USA)	2004	Lehnsahn (GER)	2016	Virginia (USA)	2017	Buchs (CH)
2015	Emsdetten (GER)	2005	Lehnsahn (GER)	2017	Buchs (CH)	2018	Buchs (CH)
2015	Florida (USA)	2006	Lehnsahn (GER)	2018	Buchs (CH)		
2015	Virginia (USA)	2007	Lehnsahn (GER)	2019	Virginia (USA)		
2015	Oregon (USA)	2008	Lehnsahn (GER)				
2016	Florida (USA)	2009	Lehnsahn (GER)				
2016	Oregon (USA)	2010	Lehnsahn (GER)				
2016	Virginia (USA)	2011	Lehnsahn (GER)				
2017	Florida (USA)	2012	Lehnsahn (GER)				
2017	Emsdetten (GER)	2013	Lehnsahn (GER)				
2017	Oregon (USA)	2014	Lehnsahn (GER)				
2017	Panevėžys (LT)	2015	Virginia (USA)				
2017	Virginia (USA)	2015	Lehnsahn (GER)				
2018	Florida (USA)	2016	Lehnsahn (GER)				
2018	Oregon (USA)	2016	Virginia (USA)				
2019	Florida (USA)	2017	Virginia (USA)				
2019	Emsdetten (GER)	2018	Lehnsahn (GER)				
2019	Virginia (USA)	2019	Lehnsahn (GER)				
2020	Florida (USA)	2019	Lehnsahn (GER)				
		2019	Virginia (USA)				
		2020	Florida (USA)				

The final analysis included all finishers, in total 969 athletes (849 men, 120 women) competing in a Double Iron, Triple Iron, Quintuple Iron, and Deca Iron ultra-triathlon. We calculated the pacing speed from lap distance and time per lap for every cycling and running lap. We excluded swimming times for the analysis because no lap times were recorded. However, evidence suggests that variations of pacing during swimming are not apparent^11^. One race had to be excluded because of missing split times (Triple Iron ultra-triathlon in Bad Blumau, Austria). We included only official finishers. Finishers with improbable speed values (i.e., cycling speed > 48 km/h and running speed > 20 km/h) were also excluded. The last races included were the Double and Triple Iron ultra- triathlon in Florida in 2020, as the other races had been canceled due to the COVID-19 pandemic.

### Statistical analyses

Data normality and homogeneity were confirmed with Kolmogorov–Smirnov and Levene’s test, respectively. Pacing variation was calculated individually as the coefficient of variation (%) between the average speed of each lap in the close circuit the athletes performed their ultra-triathlons. Each cycling lap ranges from 4 to 16.4 km and the running lap ranges from 1.4 to 3.5 km. Total distance is standard for all athletes. The performance level was defined according to the 33.3 and 66.6 percentile of overall race time for women and men. The first percentile (≤ 33.3 percentile) was named as ‘fast’, the last percentile (≥ 66.6 percentile) was named as ‘slow’, and the intermediate was named as ‘moderate’^26^. A multivariate model with two factors (two-way ANOVA) was applied for race time performance (overall, swimming, cycling, running) as the dependent variable with ‘sex’ and ‘age group’ as independent factors. Quintuple and Deca Iron ultra-triathlon were not included in the previous model because of the low number of participants across the age groups. Another multivariate model with two factors with ‘age’ and ‘sex’ as covariates (two-way ANCOVA) was applied with pacing variation (cycling and running) as dependent variable, ‘race’ and ‘performance level’ as independent factors. Partial eta squared (η_p_^2^) was calculated for the ANOVAs where the values of the effect size 0.01, 0.06, and above 0.14 were considered small, medium, and large, respectively^40^. Statistical significance was defined as *p* < 0.05. All statistical analyses were carried out with Statistical Software for the Social Sciences (IBM® SPSS v.25, Chicago, Ill, USA).

## Results

**Figure 1:**
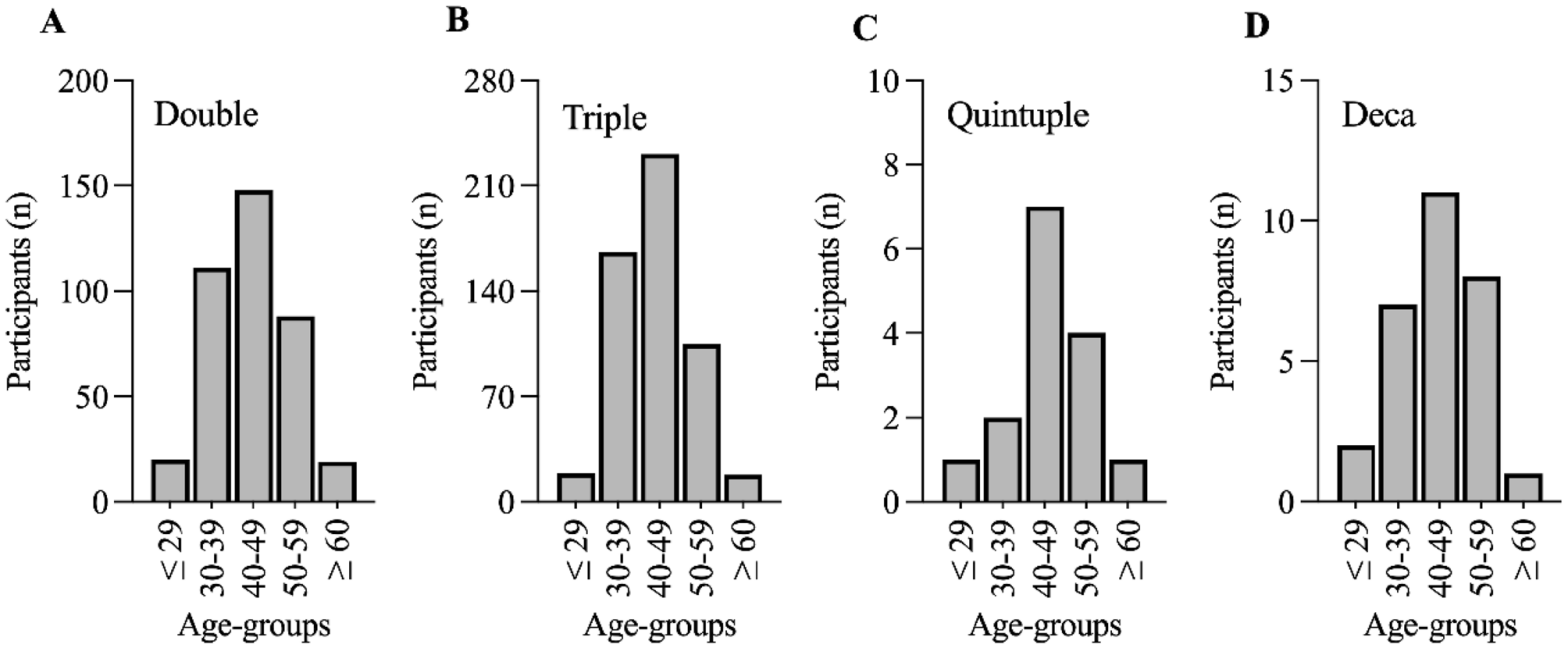
Number of participants in each age-group for Double (**A**), Triple (**B**), Quintuple (**C**), and Deca (**D**) ultra-triathlon.

The final analysis included 386 athletes competing in a Double Iron, 539 in a Triple Iron, 15 in a Quintuple Iron, and 29 in Deca Iron ultra-triathlon (*n* = 969). For all race distances, the majority of athletes were aged 40–49 years, followed by 30–39 years (Fig. 1).

**Figure 2:**
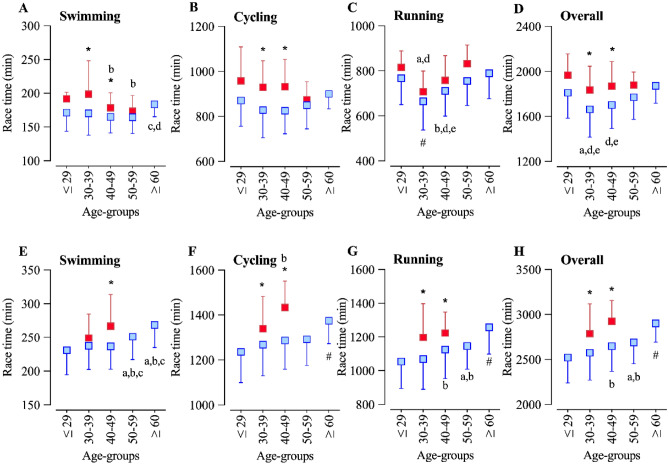
Race performance (minutes) by age groups of men and women competing in Double Iron (**A**–**D**) and Triple Iron (**E**–**H**) ultra-triathlon. *: different between sex, within the same age group, #: different from all the other age groups, a: different from age group ≤ 29 years, b: different from age group 30–39 years, c: different from age group 40–49 years, d: different from age group 50–59 years, e: different from age group ≥ 60 years.

The multivariate model for Double Iron ultra-triathlon with overall race time as the dependent variable showed that ‘age group’ was a significant factor for swimming (F = 2.48, *p* = 0.044, η_p_^2^ = 0.026), and running (F = 5.99, *p* < 0.001, η_p_^2^ = 0.060), but not for cycling or overall performance. ‘Sex’ showed significant effects for swimming (F = 4.17, *p* = 0.042, η_p_^2^ = 0.011), cycling (F = 6.36, *p* = 0.012, η_p_^2^ = 0.017), overall (F = 6.15, *p* = 0.014, η_p_^2^ = 0.016), and a borderline effect for running (F = 3.03, *p* = 0.082, η_p_^2^ = 0.008). There were no significant (*p* > 0.05) interactions for ‘age group × sex’. Pairwise comparisons showed that swimming performance for men was faster in age groups 30–39 and 40–49 years. The female age group 30–39 years was faster than older age groups, and the male age group ≥ 60 years was slower than age groups 40–49 and 50–59 years (Fig. 2A). Men’s cycling performance in age groups 30–39 and 40–49 years was faster than in the same age groups for women (Fig. 2B). Men in the age group 30–39 years ran faster than others, followed by 40–49 years (Fig. 2C). For women, athletes in the age group 30–39 years were faster than athletes in age groups ≤ 29 and 50–59 years (Fig. 2D). Women had a slower overall performance in age groups 30–39 and 40–49 years. Men in the age group 30–39 years were faster than all the others, except the age group 40–49 years (Fig. 2D).

For the Triple Iron ultra-triathlon, the multivariate model showed that ‘age group’ was a significant factor only for cycling (F = 2.66, *p* = 0.032, η_p_^2^ = 0.020). ‘Sex’ showed significant effects for cycling (F = 16.35, *p* < 0.001, η_p_^2^ = 0.030), running (F = 6.55, *p* = 0.011, η_p_^2^ = 0.012), overall (F = 13.03, *p* < 0.001, η_p_^2^ = 0.024), and a borderline effect for swimming (F = 2.91, *p* = 0.089, η_p_^2^ = 0.005). There were no significant (*p* > 0.05) interactions ‘age-group × sex’. Pairwise comparisons showed that swimming performance for men was faster in the age group 40–49 years. Men aged 50 or older swam slower than men in other age groups (Fig. 2E). Women’s cycling performance was slower than for men, and men aged 60 years or older were slower than all others (Fig. 2F). Similarly, running and overall performance were lower in women, and men aged ≥ 60 years were slower than all others, whereas athletes in the age group 30–39 were faster than in age groups 40–49 and 50–59 years (Fig. 2G and H).

**Figure 3:**
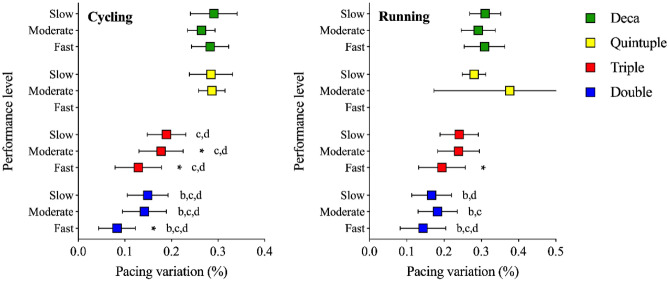
Cycling and running pacing variation in Double, Triple, Quintuple, and Deca Iron ultra-triathlon by different performance levels. *: different from all other performance levels within the same race, b: different from Triple within the same performance level, c: different from Quintuple within the same performance level, d: different from Deca within the same performance level.

The multivariate model with pacing variation as the dependent variable showed that ‘performance level’ was a significant factor for both cycling (F = 10.51, *p* < 0.001, η_p_^2^ = 0.022), and running (F = 63.63, *p* < 0.001, η_p_^2^ = 0.118). Similarly, ‘race’ was also significant for both cycling (F = 173.12, *p* < 0.001, η_p_^2^ = 0.352), and running (F = 62.97, *p* < 0.001, η_p_^2^ = 0.165). The interaction ‘performance level × race’ was significant for cycling (F = 2.31, *p* = 0.032, η_p_^2^ = 0.014) and running (F = 36.15, *p* < 0.001, η_p_^2^ = 0.185). Pairwise comparisons showed that pacing variation was lower for faster athletes cycling in Double and Triple Iron ultra-triathlon and running in Triple Iron ultra-triathlon. Cycling pacing variation in Double Iron ultra-triathlon was the lowest across all performance levels. It was lower in Triple Iron ultra-triathlon than in Quintuple and Deca Iron ultra-triathlon. For running, all performance levels of Double Iron ultra-triathlon showed a lower pacing variation than in the Triple Iron ultra-triathlon. Moderate and fast athletes were also different from the Quintuple Iron ultra-triathlon and slow and fast athletes were different from the Deca Iron ultra-triathlon (Fig. 3).

## Discussion

This study intended to investigate the trends in pacing and the effects of performance level, sex and age in ultra-triathlons with the primary hypothesis of a positive pacing strategy in Double and Triple ultra-triathlon and an even pacing strategy in Quintuple and Deca ultra-triathlon.

The first important finding was a positive pacing strategy applied in all distances confirming our hypothesis for Double and Triple ultra-triathlon. This is consistent with other studies, where positive pacing strategies were observed in long-distance events. In 2014, the top 100 Ironman® finishers adopted a positive pacing strategy in most races^15^. Two studies investigating 6 and 23 finishers competing in Deca Iron ultra-triathlon showed that split and overall race times increased linearly across the ten days^20,41^. However, several studies showed athletes adopting an even pacing strategy^41–43^. Male Triple Deca Iron ultra-triathletes competing for 30 days finishing an Ironman® distance triathlon daily could maintain their performance^5^. Additionally, a male triathlete competing for 33 days and finishing an Ironman® distance triathlon daily was able to maintain his performance in cycling, running, and overall race times^42^. The best performing female in Quintuple and Deca Iron ultra-triathlon applied an even pacing strategy during her two world record races^43^. A potential explanation for the consistent pacing could be her background as an elite cyclist and her previous experience^43^. Another important aspect is that we compared continuous races instead of one per day races. In one-a-day events, the change between disciplines might give enough rest to allow a steady pace throughout the race.

A second important finding was the difference in pacing variation. We hypothesized that slower finishers would apply more variable pacing. Faster finishers showed a significantly lower variation in cycling pacing in Double and Triple Iron ultra-triathlon and running pacing in the Triple Iron ultra-triathlon, confirming our hypothesis. Although not significant, faster athletes running in a Double Iron ultra-triathlon also showed the lowest pacing variation. This is most likely explained by more ‘moderate’ athletes being split into three even groups. A recent study regarding elite triathletes showed that a lower variability in race pacing during a10-km run also reflected more successful run times^44^. Keeping a low pacing variation seems to be the appropriate strategy for most distances and disciplines up to a Triple Iron ultra-triathlon.

Pacing variation is also different within disciplines. The variation in pace was lower in cycling than in running. This result corroborates with data of the world’s best female ultra-triathlete in Quintuple and Deca Iron ultra-triathlon where she showed a higher pacing variation in running than in cycling^43^. Drinking and eating in such events is often reported to be harder in running than in cycling, as a quick stop in running reduces the speed to zero, whereas in cycling the speed drops slowly even when one stops pedaling^43^.

In the longer race distances, the pacing variation increased. There were no significant changes in pacing variation between the different performance levels in the Quintuple and Deca Iron ultra-triathlon. These contrasting findings might be due to the low participation number in Quintuple and Deca Iron ultra-triathlon. There is no systematic research comparing pacing variation in ultra-triathlon to this day. With increasing distance, exercise economy seems harder to maintain or limited (i.e., stops for sleeping and nutrition), resulting in a more significant pacing variation in longer distances^45^.

A further important finding was that men paced faster than women confirming our hypothesis of different pacing between men and women. Women in age groups 30–39 and 40–49 years were overall significantly slower than men. The difference between sexes increased with increasing race distance for swimming, running, and overall race time. However, there was no significant difference in swimming time between men and women in age group 30–39 years in Triple Iron ultra-triathlon. This finding is consistent with an analysis of open-water ultra-distance swimmers from 5 to 25 km, where the difference between sexes was lowest in 10 km compared to 5 km and 25 km^46^. For ultra-swimming, women seemed to achieve a similar or even better performance than men^34,47^.

The difference between sexes in endurance performance is primarily caused by physiological differences in VO_2_ max^48^ and anthropometric characteristics such as skeletal muscle mass and body fat^49^. It has been shown that female ultra-runners have lower skeletal muscle mass and a higher percentage of body fat than male ultra-runners, which may disadvantage women in ultra-running performance^50^.

Regarding age, most participants were in age group 40–49 years, followed by the age group 30–39 years for all race distances. We hypothesized that ultra-triathletes would reach peak performance at a higher age compared to Ironman® triathletes. We could confirm this in the Double Iron ultra-triathlon, where men achieved the best performances overall in age groups 30–39 and 40–49 years while in Ironman® triathlon, peak performance for men was reported to be at 32.2 ± 1.5 years^51^. Regarding running performance in Double Iron ultra-triathlon, athletes in the age group 30–39 years were the fastest. Several studies showed that endurance and ultra-endurance performance in disciplines such as Ironman® triathlon and ultra-marathon appeared to be maintained until the age of 35–40 years, followed by modest decreases until 50 years of age and a progressive decrease in performance thereafter^37,52,53^.

Women in Double Iron ultra-triathlon showed no difference in overall performance across all age groups. In swimming, athletes in the age group 40–49 and 50–59 years were significantly faster than athletes in the age group 30–39 years. In a study regarding the age-related performance decline in Ironman® triathlon, the decline in swimming performance already started in the age group 25–29 years for both women and men^3^. A possible explanation for our finding could be the low participation number of women in long endurance events such as Iron ultra-triathlon or ultra-marathons. A study investigating participation trends in Triple Iron ultra-triathlon from 1988 to 2011 showed a stable participation rate of ∼8%^54^. In other ultra-endurance distances such as 100-km ultra-marathons, female participation was higher at ∼13% but still very low^50^. Consequently, the athletes competing have a good estimation of the extent of their performance due to their accumulated experience and therefore applied a pacing strategy that was right and correct. In addition, athletes might stop competing after an injury, so older athletes result from a further selection process that could be described as ’survival of the fittest^3^. Another factor for the increase in performance in women in older age groups could be that the motivation in achieving best times has more importance for women competing in these races^55^.

In contrast to our hypothesis, in Triple Iron ultra-triathlon, athletes in the age group 30–39 years were faster than athletes in the age group 40–49 years, suggesting a similar age of peak performance as reported in Ironman® triathlon for men^51^. A previous study investigating both Triple Iron ultra-triathletes and Deca Iron ultra-triathletes showed that athletes were able to maintain their best performances for ages comprised between 25 and 44 years, independent of the race distance^39^. To summarize, athletes in only one age group (10-years interval) between 40 and 49 years might hide an age-related decline in the age group 44–49 years (e.g., sampling bias). Peak performance might still be achievable between 40 and 44 years. There were no significant differences between the age groups in women participating in the Triple Iron ultra-triathlon. This is primarily explained by the low participation number of women. Quintuple and Deca Iron ultra-triathletes were not included because of the low number of participants across the age groups.

Age-related declines in endurance and ultra-endurance performance have been well described in the literature for running^56^, cycling^57^, swimming^58^ and for triathlon^37,59^. The duration of a triathlon race exerts an important influence on the age-related changes in triathlon performance^60,61^.

The age-related performance decline in Ironman® triathlon starts with 25–29 years in swimming for both women and men and in the age group 35–39 years for men respective age 30–34 years for women in cycling, running and overall performance^3^. We hypothesized that the age-related declines would occur later in the longer distances (e.g., Quintuple and Deca Iron ultra-triathlon) compared to the shorter distances (e.g., Double and Triple Iron ultra-triathlon). We could confirm our hypothesis for overall performance as in Double Iron ultra-triathlon the decline started with age group 50–59 years. However, our hypothesis could not be confirmed for the Triple Iron ultra-triathlon where the age group 40–49 years was significantly slower than 30–39 years. In swimming, there were no significant differences for men until an age-related decline in the age group 50–59 years in Double Iron ultra-triathlon and age > 60 years in Triple Iron ultra-triathlon. No significant differences were observed in cycling times for all age groups. The age-related decline for running started for both Double and Triple Iron ultra-triathletes in the age group 40–49 years. This is consistent with other studies, finding that the age-related decline in swimming and cycling was less pronounced^39,59,62^. The decrease in running performance for men in Triple Iron ultra-triathlon seems to be much more impactful on overall performance than in Double Iron ultra-triathlon. Quintuple and Deca Iron ultra-triathlon were not included because of the low number of participants across the age groups.

### Strength, weakness, limitations and implications for future research

The strength of this study is the large data set including all finishers of ultra-triathlon races held worldwide between 2004 and 2020, with a total of 849 male and 120 female finishers in 46 ultra-triathlons. However, some races might not have been documented on the official IUTA website. In earlier years, lap times were not recorded electronically. Another strength of this study is its novelty, as it is the first one containing a detailed analysis of the pacing variation within the laps of the races. A weakness is that we were not able to consider environmental conditions^63–65^ and individual factors such as anthropometric^49^ and nutritional characteristics^66,67^ and previous experience^49^. Besides environmental conditions, other race characteristics can influence pacing, such as elevation in cycling and running, swimming in open water (sea, lake) or a pool. Another weakness is the low participation number of women and the low participation numbers in Quintuple and Deca Iron ultra-triathlon. This is due to the low number of races held for these distances. We did not include non-finishers, so including those might give further insight into which pacing strategies result in failure. Future studies would need to include more female participants and a higher number of participants across all age groups in Quintuple and Deca ultra-triathlon.

## Conclusion

Ultra-triathletes adapted a positive pacing strategy, i.e., speed decreased over the duration of the race. In addition, faster athletes show less variation in pacing then moderate and slower athletes in Double and Triple Iron ultra-triathlon. Pacing variation differed between the disciplines, with the lowest variation in cycling. Consequently, ultra-triathletes should be advised to adopt less variable pacing while maintaining a positive pacing strategy. For professional athletes, an even pacing strategy might be achievable. Men pace faster than women. The sex difference increased with increasing race distance for swimming, running, and overall race time. It seems unlikely that women will outperform men with increasing distance.

The best age for men in Double Iron ultra-triathlon is between 30 and 49 years and in Triple Iron ultra-triathlon between 30 and 39 years. The age-related performance decline in the age group 40–49 years is due to the lower running performance. This leads to a significant decrease in overall performance in the Triple Iron ultra-triathlon. Training should emphasize that finding by focusing more on improvement in running times.

## Data Availability

The athletes' data was downloaded from the official IUTA website (www.iutasport.com/).
